# Structural stability and mechanism of compression of stoichiometric B_13_C_2_ up to 68GPa

**DOI:** 10.1038/s41598-017-09012-8

**Published:** 2017-08-21

**Authors:** Irina Chuvashova, Elena Bykova, Maxim Bykov, Volodymyr Svitlyk, Leonid Dubrovinsky, Natalia Dubrovinskaia

**Affiliations:** 1 Material Physics and Technology at Extreme Conditions, Laboratory of Crystallography, University of Bayreuth, D-95440 Bayreuth, Germany; 2 Bayerisches Geoinstitut, University of Bayreuth, D-95440 Bayreuth, Germany; 3 Photon Sciences, Deutsches Elektronen-Synchrotron (DESY), D-22607 Hamburg, Germany; 4 European Synchrotron Radiation Facility, BP 220, F-38043 Grenoble Cedex, France

## Abstract

Boron carbide is a ceramic material with unique properties widely used in numerous, including armor, applications. Its mechanical properties, mechanism of compression, and limits of stability are of both scientific and practical value. Here, we report the behavior of the stoichiometric boron carbide B_13_C_2_ studied on single crystals up to 68 GPa. As revealed by synchrotron X-ray diffraction, B_13_C_2_ maintains its crystal structure and does not undergo phase transitions. Accurate measurements of the unit cell and B_12_ icosahedra volumes as a function of pressure led to conclusion that they reduce similarly upon compression that is typical for covalently bonded solids. A comparison of the compressional behavior of B_13_C_2_ with that of α–B, γ–B, and B_4_C showed that it is determined by the types of bonding involved in the course of compression. Neither ‘molecular-like’ nor ‘inversed molecular-like’ solid behavior upon compression was detected that closes a long-standing scientific dispute.

## Introduction

Boron carbide B_4_C was discovered^1^ in 1858, and its crystal structure was first established^2^ in 1934. It belongs to icosahedral boron compounds, a family of crystalline solids with crystal structures based on various arrangements of B_12_ icosahedra, which are considered to be some kind of B_12_ “molecules”. In such solids chemical bonding has been rationalized in terms of polycenter bonds on the B_12_ *closo* **-**cluster (boron icosahedron) and two-electron-two-center (*2e2c*) and two-electron-three-center (*2e3c*) bonds between the clusters^3^. According to the Wade-Jemmis rule^4–6^ 26 out of 36 valence electrons of a B_12_ cluster are accommodated in 13 molecular-orbital-like bonding orbitals to form the cluster. This leaves 10 electrons for external bonding using 12 equivalent external bonding orbitals, thus creating an electron deficiency in the B_12_ cluster. For a long time, this ‘electron deficiency’ of the intraicosahedral bonding relative to the intericosahedral one was expected to make the icosahedra more compressible compared to the unit cell, contrary to what is known for typical molecular solids. For this reason the icosahedral boron-rich solids were understood as “inverted-molecular” solids^7^. This gave the origin to the problem of the ratio of the rigidity of the B_12_ *closo* **-**cluster and the unit cell and the mechanism of compression in icosahedral boron-rich solids that had to be proven experimentally.

The first work, which aimed to shed light into the mechanism of compression of icosahedral boron-rich solids in general and boron carbide in particular, was a high-pressure powder neutron diffraction study of boron carbide (B_4_C) to 11 GPa conducted by Nelmes and co-authors^8^. By the linear fit of the experimental ‘pressure *versus* volume’ (P-*V*) data obtained for both the icosahedron and the unit cell of B_4_C, the bulk moduli of the icosahedron and the entire structure were determined to be 169(6) GPa and 199(7) GPa, respectively. This led to conclusion that the icosahedron is 23(4) % more compressive than the unit cell of boron carbide, which thus behaved as an “inverted-molecular” solid^8^.

An experimental evidence that icosahedral boron materials on compression may not behave as “inverted-molecular” solids came from the single-crystal X-ray diffraction data to 65 GPa obtained for the high-pressure boron allotrope, γ-B^9^. The bulk modulus of the B_12_ icosahedron was determined to be 285(6) GPa, whereas the bulk modulus of the entire γ-B structure was found to be 227(3) GPa^9^.

A new insight into the situation in boron carbide, compared to the very first measurements^8^, was given due to a compressibility study of “nearly stoichiometric boron carbide B_4_C”^10^. Single crystals of B_4_C were investigated using synchrotron X-ray diffraction in a diamond anvil cell to 74 GPa. The structure of B_4_C was understood as consisting of B_11_C icosahedra interconnected by the C-B-C chains. The parameters of the equation of state (EoS) of boron carbide were *K*_300_ = 243 (6) GPa (*K′* = 3.6 (2)) or 236 (8) at fixed *K′* = 4 (*K*_300_ is the bulk modulus, and *K′* is its pressure derivative; the zero pressure unit cell volume was fixed on the ambient pressure experimental value). Dera *et al*.^10^ pointed out that the “icosahedron volume compression did not follow a typical EoS functional behavior” and they did not calculate the bulk modulus of the B_11_C icosahedron. Nevertheless, they reported that the B_11_C icosahedron showed a 13% volume reduction, which was smaller than that of the unit cell volume (18%). The general conclusion was that B_4_C behaves as a molecular solid^10^ that is in accordance with the results obtained for γ-B^9^.

Optical properties and evolution of Raman modes of the same B_4_C samples, which were characterized and studied using single-crystal X-ray diffraction in ref. 10, were also investigated up to 70 GPa by the same research group^11^. Based on their spectroscopy data, the authors^11^ reported high-pressure phase transition in boron carbide at 40 GPa, although, according to Dera *et al*.^10^, no signatures of structural phase transitions were observed in B_4_C by high-pressure XRD studies up to 70 GPa^10^. Verifying these mutually contradictive reports on boron carbide is not only scientifically important on itself, but it is additionally justified in connection to the commercial value of this unique material. Boron carbide is a very hard and, at the same time, lightweight material for applications in personal security (bullet-proof vests)^3^. It possesses the highest Hugoniot elastic limit of ceramic materials (ca. 17–20 GPa), i.e. the maximum uniaxial dynamic stress that the material can withstand elastically, surpassing all its denser competitors such as silicon carbide and alumina by a factor of 2 (ref. 12). However, it fails just above the Hugoniot elastic limit and the possible source of failure could be clarified through high-pressure experiments. Thus, establishing the mechanism of compression of boron carbide, clarifying its mechanical properties and limits of its stability under loading, are of both scientific and practical interest.

Here we report high-pressure investigations of stoichiometric boron carbide B_13_C_2_ using high-pressure single-crystal X-ray diffraction up to 68 GPa. Single crystals of B_13_C_2_, which we study here, were characterized in detail in previous work of our group^13^. It was established that B_13_C_2_ is fully ordered and stoichiometric, and carbon atoms occupy a single position (at the ends of the C-B-C chains)^13^. In the present work we could track all changes of the crystal structure, atomic positions, and bond lengths with the high accuracy up to 68 GPa. This enabled us to establish the equations of state of both the B_13_C_2_ crystal and the B_12_ icosahedron. As the B_12_ icosahedra in the stoichiometric boron carbide B_13_C_2_ do not contain any carbon atoms, contrary to all previously investigated boron carbides^8, 10, 14–24^, we could compare the compressional behavior of B_12_ *closo*-clusters in B_13_C_2_ and boron allotropes α-B and γ-B and conclude not only regarding the ratio of the rigidity of the B_12_ *closo*-cluster and the unit cell in these materials, but also concerning the mechanism of compression of boron-rich icosahedral solids.

## Results

### The equations of state of B_13_C_2_ and B_12_ icosahedra

The single-crystal X-ray diffraction data for B_13_C_2_ obtained at variable pressure and some experimental details are presented in Table 1. The quality of the data allowed the refinement of both the lattice parameters and atomic coordinates (Supplementary Table S1). The quality of the structural refinement was good up to the highest pressure achieved that gives evidence that in the course of compression the crystals were maintained in quasihydrostatic environment.

**Table 1:** Crystallographic details of B_13_C_2_ at variable pressure. Formula: B_13_C_2_; Crystal System: Trigonal; Z = 3; Space group: *R* $\mathop{{\boldsymbol{3}}}\limits^{\bar{}}$ *m*, crystal size 10 × 10 × 15 µm^3^ (crystal 1) and 20 × 15 × 10 µm^3^ (crystal 2, designated as “cr2”).

P, GPa	*a*, Å	*c*, Å	*V*, Å^3^	R_int_*	R_1_[I > 3σ(I)]*	N_m_**	N_i_**
0.00010 (1)^13^	5.5962 (3)	12.0661 (7)	327.25 (3)	0.0197	0.0227	505	440
4.0 (5)	5.5653 (6)	11.9811 (11)	321.37 (6)	0.093	0.0755	272	155
10.0 (5)	5.5321 (9)	11.898 (2)	315.33 (9)	0.024	0.0762	221	150
20 (1)	5.4708 (5)	11.7296 (9)	304.03 (4)	0.025	0.0577	362	149
23 (1)	5.4597 (12)	11.6932 (19)	301.86 (11)	0.024	0.1096	202	148
30 (1) cr2	5.4134 (8)	11.5589 (18)	293.35 (8)	0.023	0.0659	291	123
35 (1) cr2	5.383 (4)	11.472 (8)	287.9 (3)	0.030	0.0595	316	122
43 (1) cr2	5.353 (4)	11.413 (8)	283.2 (4)	0.028	0.0617	268	117
49 (1) cr2	5.335 (4)	11.334 (8)	279.3 (3)	0.024	0.0584	247	119
56 (1) cr2	5.3007 (9)	11.257 (2)	273.91 (9)	0.024	0.0617	236	121
65 (1)	5.2683 (10)	11.1812 (16)	268.75 (8)	0.055	0.0816	184	100
68 (1) cr2	5.2529 (50)	11.1267 (10)	265.88 (4)	0.024	0.0645	200	109

*R_int_ and R_1_[I > 3 σ(I)] relate to data collection and structure refinement, respectively.

**N_m_ is the number of measured unique reflections; N_i_ - the number of unique reflections with I > 3σ(I).

**Figure 1:**
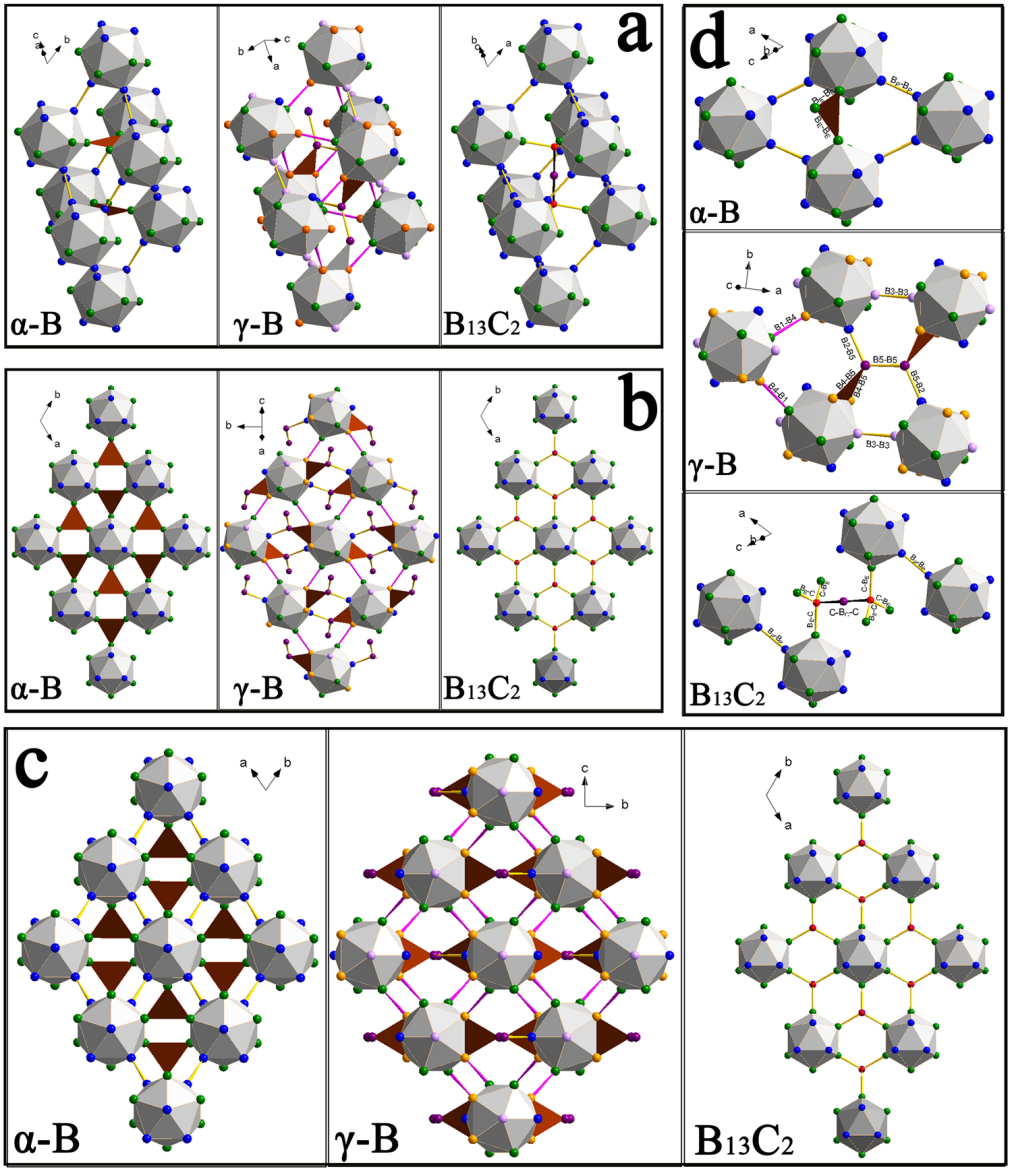
Crystal structures of α-B, γ-B and B_13_C_2_. (**a**) Rhombohedral cells of α-B and B_13_C_2_ compared to the elemental rhombohedron which can be selected in the structure of γ-B. (**b**) Single layers of the cubic closest packing (*ccp*) of icosahedra in the structures of α-B, γ-B. and B_13_C_2_. The view is perpendicular to the layers: for α-B and B_13_C_2_ the direction of the view coincides with the *c*-axis of the trigonal unit cell (hexagonal settings), for γ-B it coincides with the *c´* direction (see text). (**c**) The packing of icosahedra shown in projection along the axes of the rhombohedral cells for α-B and B_13_C_2_ and along the *a*-axis for γ-B. (**d**) Fragments of the structures of α-B, γ-B and B_13_C_2_ showing different types of bonds in the corresponding structures (see text for details). In all structure drawings, atoms in crystallographically independent positions are marked in different colors: for α-B and B_13_C_2_ B_P_ are blue, B_E_ are green; for B_13_C_2_ carbon and boron atoms of the C-B-C chains are B_C_ (violet) and C (red). For γ-B B1 are blue, B2 are green, B3 are purple, B4 are orange, B5 are violet. Different types of bonds are shown in different colors: *2e2c* bonds are yellow, *1e2c* bonds are magenta, *2e3c* bonds are brown triangles, *3e3c* bonds are black.

In the structure of boron carbide B_12_ icosahedra are located in the corners of the rhombohedral cell, and intericosahedral three-atom C-B-C linear chains are oriented along its body diagonal (Fig. 1a, right). As seen in Fig. 1, the structure of B_13_C_2_ is very similar to those of α-B and γ-B, which can be described in terms of a cubic closest packing (*ccp*) of spheres, where B_12_ icosahedra play the role of “spheres” (Fig. 1a,b). The unit cell parameters of B_13_C_2_ in hexagonal setting are *a* = 5.5962 (3) Å, *c* = 12.0661 (7) Å, (space group R $\mathop{3}\limits^{\bar{}}$ m), as determined in ref. 13. Boron atoms in the crystal structure of B_13_C_2_ (Fig. 1) occupy three crystallographically independent positions (B_P_ , B_E_, and B_C_) and the forth position is occupied by carbon atoms in the C-B-C chains^13^. In the present paper we adopt the nomenclature introduced by ref. 13: boron atoms forming the boron icosahedra are labeled as B_P_ (polar positions) and B_E_ (equatorial positions), B_C_ designates the boron atom in the center of the C-B-C chain (Fig. 1).

**Figure 2:**
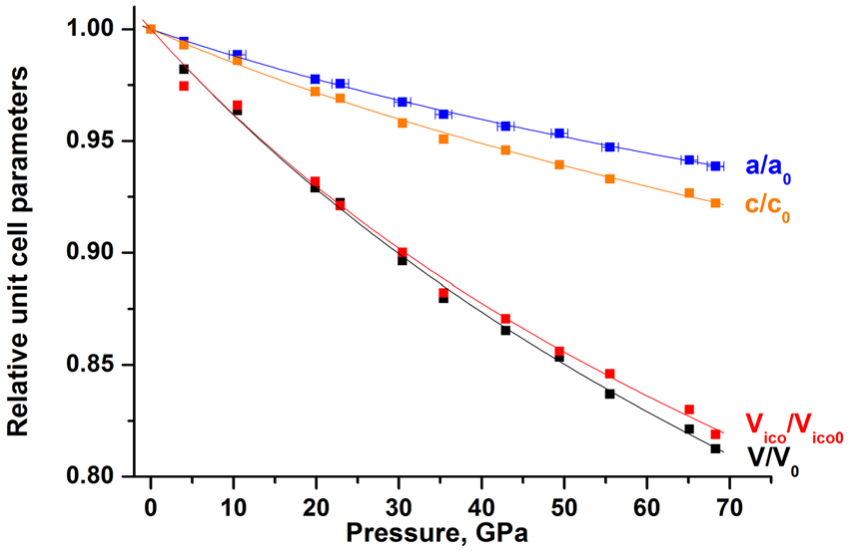
Pressure-dependent evolution of the relative lattice parameters and the relative unit cell and B_12_ icosahedron volumes for B_13_C_2_. Continuous lines show the fit of the corresponding data with the third-order Birch-Murnaghan equation of state.

In this study, all observed reflections perfectly match the B_13_С_2_ structure up to the highest pressure reached. Our X-ray diffraction data did not reveal any indication of a phase transition. All the unit cell parameters smoothly decrease on compression (Table 1). Figure 2 presents the dependence of the relative unit cell parameters (*a/a*_0_ and *c/c*_0_) and the relative unit cell (*V/V*_0_) volume of B_13_C_2_ on pressure up to 68 GPa. As seen (Fig. 2), the structure of B_13_С_2_ is slightly more compressible along the *c* direction.

The whole experimental volume-pressure data set was fitted using the third-order Birch-Murnaghan (3BM) equation of state that gave the following EoS parameters: *V*_*0*_ = 327.25 (4) Å^3^, *K*_*300*_ = 239 (7) GPa and *K′* = 3.2 (3) (*V*_*0*_ is the zero pressure unit cell volume, *K*_*300*_ is the bulk modulus, and *K′* is its pressure derivative) (Table 2). The fit with the fixed *K′* = 4 resulted in the bulk modulus of *K*_*300*_ = 222 (2) GPa, being lower than that with the free *K′*.

**Table 2:** Parameters of the equations of state of B_13_С_2_, B_4_C, α-B, and γ-B obtained on the basis of single-crystal XRD in comparison with those of icosahedra. Volume reduction of the unit cells and icosahedra on compression was calculated for the pressure range from ambient to 60 GPa.

Material		Reference	*K*_*300*_, GPa	*K′*	*K*_*ico*_, GPa	*K′*_*ico*_	Volume reduction, %	
							Unit cell	Icosahedron
boron carbide	*B*_13_ *C*_2_	*Present study*	*239 (7)*	*3.2 (3)*	*239 (23)*	*3.8 (8)*	*18.7*	*18.1*
	B_4_C	Dera *et al*. (2014)^10^	243 (3)	3.6 (2)			18	13
α-B		Chuvashova *et al*. (2017)^25^	224 (7)	3.0 (3)	273 (12)	4 (fixed)	18.0	14.5
γ-B		Zarechnaya *et al*. (2010)^9^, P < 40 GPa	227 (3)	2.5 (2)	285 (6)	1.8 (3)	18.9	16.9

The evolution of the relative volume of the B_12_ icosahedron (*V*_*ico*_ */V*_*ico0*_) with pressure is presented in Fig. 2. It is very similar to that of the unit cell volume. The whole experimental data set of the icosahedra volumes *versus* pressure (Table 3) was fitted using the 3BM EoS. The EoS parameters were obtained as follows: *V*_*ico0*_ = 12.50 (3) Å^3^, *K*_*ico*_ = 239 (23) GPa, and *K*_*ico*_ *′ *= 3.8 (8) (*V*_*ico0*_ is the zero pressure icosahedron volume, *K*_*ico*_ is the bulk modulus of icosahedra, and *K*_*ico*_ *′* is its pressure derivative). The fit with the *K*_*ico*_ *′* = 4 (fixed) results in a very close value of *K*_*ico*_ = 234 (7) GPa. A comparison of the bulk moduli found for the B_13_С_2_ crystal and the B_12_ icosahedron (Table 2) shows that the bulk B_13_С_2_ and the icosahedron have the similar rigidity.

**Table 3:** The B_12_ icosahedron volume and bond lengths in boron carbide at variable pressure. “cr2” designates crystal 2. B_P_ , B_E_ and B_C_ are designations from Mondal *et al*.^13^; b1 through b7 are designations from Dera *et al*.^10^.

P, GPa	*V*_*ico*_, Å^3^	Bonds involving CBCs		Inter-cluster bond	Intra-cluster bonds			
		B_E_-C (b7)	B_C_ –C (b1)	B_P_-B_P_ (b2)	B_P_–B_P_ (b6)	^1^B_P_–B_E_ (b4)	^2^B_P_–B_E_ (b5)	B_E_–B_E_ (b3)
0.00010 (1)^13^	12.501	1.6037 (2)	1.4324 (5)	1.7131 (4)	1.8053 (4)	1.7997 (4)	1.7848 (5)	1.7590 (3)
4.0 (5)	12.1908	1.5980 (17)	1.4246 (48)	1.7112 (32)	1.7825 (26)	1.7826 (28)	1.7786 (33)	1.7480 (23)
10.0 (5)	12.0831	1.5828 (16)	1.4194 (36)	1.6874 (31)	1.7769 (26)	1.7817 (28)	1.7686 (33)	1.7394 (22)
20 (1)	11.6379	1.5653 (21)	1.4098 (35)	1.6649 (30)	1.7523 (26)	1.7605 (27)	1.7474 (30)	1.7177 (21)
23 (1)	11.5144	1.5627 (41)	1.4102 (70)	1.668 (62)	1.7406 (52)	1.7525 (53)	1.7469 (58)	1.7147 (41)
30 (1) cr2	11.2751	1.5476 (15)	1.3963 (35)	1.6346 (26)	1.7420 (17)	1.7412 (21)	1.7232 (31)	1.6989 (17)
35 (1) cr2	11.0331	1.5418 (11)	1.3904 (34)	1.6234 (26)	1.7274 (17)	1.7292 (22)	1.7130 (28)	1.6855 (16)
43 (1) cr2	10.8796	1.5321 (11)	1.3890 (34)	1.6113 (26)	1.7146 (17)	1.7207 (22)	1.7109 (28)	1.6778 (16)
49 (1) cr2	10.7212	1.5222 (11)	1.3871 (34)	1.5915 (24)	1.7057 (17)	1.7160 (21)	1.6990 (28)	1.6667 (16)
56 (1) cr2	10.5721	1.5159 (11)	1.3790 (34)	1.5821 (24)	1.6994 (17)	1.7064 (22)	1.6921 (28)	1.6594 (16)
65 (1)	10.4294	1.5016 (15)	1.3775 (45)	1.5689 (34)	1.6890 (33)	1.6974 (35)	1.6867 (40)	1.6545 (29)
68 (1) cr2	10.2436	1.498 (2)	1.3719 (33)	1.5757 (30)	1.6778 (25)	1.6876 (27)	1.6735 (31)	1.6477 (21)

### Evolution of the bond lengths on compression of B_13_С_2_

Recent experimental electron-density study using low-temperature high-resolution single-crystal synchrotron X-ray diffraction data^13^ clarified the bonding situation in the stoichiometric boron carbide B_13_С_2_. In the present work, we investigated single crystals from the same batch. For consistency, we adopt here the notations of bonds introduced in ref. 13.

There are seven distinct bonds in the structure of boron carbide (Table 3), which get into three groups^13^ (see Fig. 1d, bottom, for bonds notations): intra-cluster polycentral bonds (B_P_–B_P_ , B_E_–B_E_, ^1^B_P_–B_E_ and ^2^B_P_–B_E_); inter-cluster bonds (B_P_–B_P_), which connect atoms in the polar sites (B_P_) of the neighboring icosahedra; and bonds involving C-B-C chains (C–B_E_ and C– B_C_).

**Figure 3:**
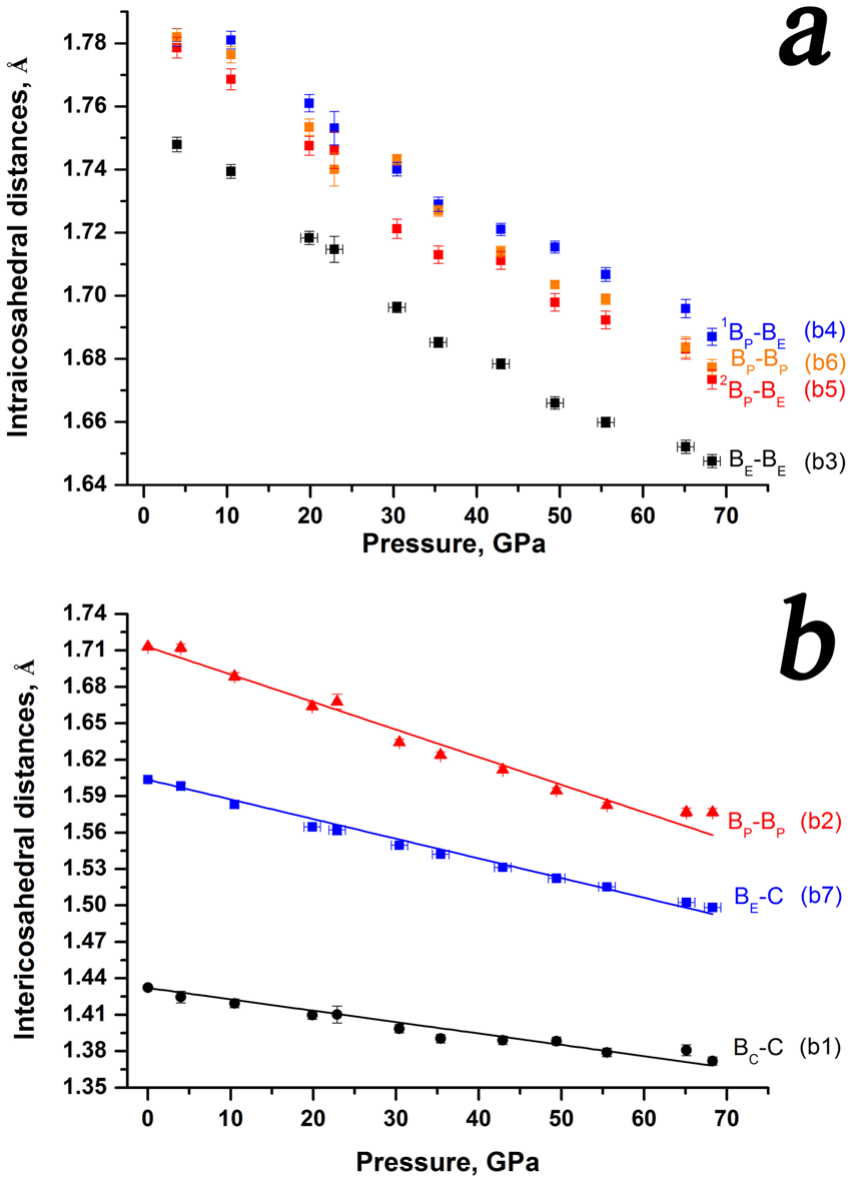
Pressure-dependent evolution of bonds of B_13_C_2_. (**a**) Intraicosahedral bonds; (**b**). Intericosahedral bonds. Bonds designations are according to Mondal *et al*.^13^, corresponding notations from Dera *et al*.^10^ (b1 through b7) are given in brackets to make it easier to compare.

Single-crystal X-ray diffraction data, collected at eleven pressure points in the interval from 4 GPa to 68 GPa, allowed us to follow changes in the length of each of the seven bonds in the structure of boron carbide B_13_С_2_ (Table 3). All bonds gradually shorten under compression (Fig. 3); their pressure-dependent evolution does not show any anomalies (Fig. 3).

## Discussion

As mentioned in the introduction, bulk compressibility of boron carbide compared to compressibility of icosahedra has been a matter of debate. Nelmes *et al*.^8^ reported the crystal structure to be more rigid than the icosahedron cluster, whereas Dera *et al*.^10^ observed an opposite relation.

Our results have shown that the rigidity of the crystal structure of stoichiometric boron carbide B_13_C_2_ is similar to that of the B_12_ icosahedron. Within the standard uncertainty, we found the bulk moduli of the bulk material and the icosahedra to be similar: *K*_*300*_ = 239(7) GPa (*K′* = 3.2(3)) *versus* *K*_*ico*_ = 239(23) (*K*_*ico*_ *′* = 3.8(8)) (with the fixed *K′ *= 4, *K*_*300*_ = 222(2) GPa *versus* *K*_*ico*_ = 234(7) GPa). The volume reduction of the unit cell of B_13_C_2_ and the volume reduction of the icosahedron in the pressure interval between ambient and 60 GPa were calculated to be also similar (18.7% *versus* 18%, respectively); the difference is less than 1% and within the uncertainty. The bulk modulus obtained by Dera and co-authors^10^ for the crystals of B_4_C is in a very good agreement with our result: their 3BM EoS parameters are *K*_*300*_ = 243 (3) GPa, *K′* = 3.6(6), and *V*_*0*_ = 330.59(5) Å^3^ (*V*_*0*_ was fixed at experimental value obtained at ambient conditions); with the fixed *K′ *= 4, they got *K* = 236(8) GPa^10^. The volume reduction of the unit cell of B_4_C (18%)^10^ matches very well to what we obtained for B_13_C_2_. But interestingly, the volume reduction of the icosahedron in B_4_C in the same pressure interval (between ambient and 60 GPa) in ref. 10 appeared to be different (13%) (see Table 2).

To clarify a reason of the apparent difference in the ratio of the rigidity of the unit cell and icosahedra found for B_13_C_2_ and B_4_C^10^, we first compared the icosahedron and the unit cell volume evolution with pressure in the stoichiometric boron carbide B_13_C_2_ and boron allotropes α-B^25^ and γ-B^9^. In the structures of each of these materials, icosahedra (B_12_) are built of exclusively boron atoms. Table 2 demonstrates a remarkable observation: in the same pressure range (from ambient up to 60 GPa), the unit cell volume reductions for all these materials are similar (ca. 18% within less than 1% deviation). The B_4_C, containing B_11_C icosahedra, is not an exception (18%)^10^. However, the icosahedra volume reductions are all different and reduce in the raw B_13_C_2_ (18.1%), γ-B (16.9%), α-B (14.5%), and B_4_C (13%). This observation is striking enough and desires an explanation through an insight into the compressional behavior of individual bonds in these solids.

**Figure 4:**
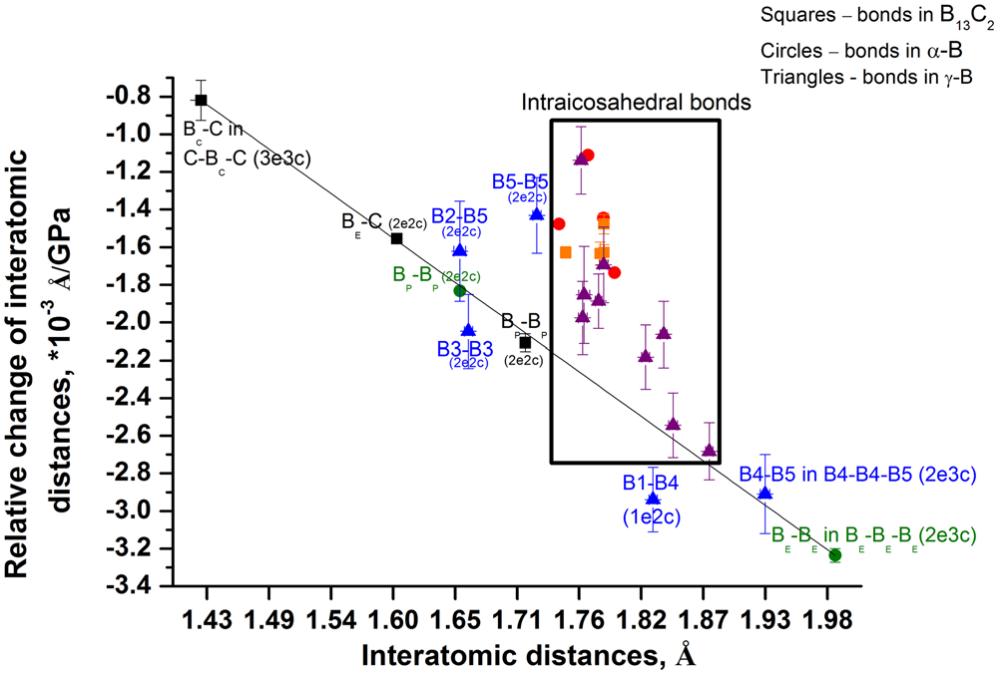
Relative change of interatomic distances for α-B, γ-B and B_13_C_2_ single crystal plotted against their length at lowest pressure as revealed by *in situ* single-crystal X-ray diffraction. Circles stay for bonds in α-B, triangles for γ-B, and squares for B_13_C_2_. Intraicosahedral bonds are outlined by the black rectangular; red, purple and orange symbols correspond to α-B, γ-B and B_13_C_2_, respectively. Intericosahedral bonds and those involving B-B dumbbells and C-B-C chains are shown in green, blue, and black colors for α-B, γ-B and B_13_C_2_, respectively.

To visualize the difference in the rates of changes of the bond length and compare boron carbide B_13_C_2_ with α-B and γ-B, experimentally obtained data for the relative changes of the bond lengths (*l*_*P*_ */l*_*P0*_) *versus* pressure was linearly fitted for all the bonds (*l*_*P*_ is the length of the bond at pressure P; *l*_*P0*_ is the length of this bond at P_0_ = 4.0 GPa, the first pressure point available in our experiment in the DAC). We plotted calculated “line slopes” against corresponding interatomic distances *l*_*P0*_ (Fig. 4) at the lowest pressure, similarly to how it was done for characterization of the bond lengths’ change under pressure for various boron-rich compounds^26^ and α-B^25^.

As seen in Fig. 4, all the points corresponding to the inter-cluster bonds (C–B_C_, C–B_E_, and B_P_–B_P_) in B_13_C_2_ (black squares) lie on one line, and the rate of their compression depends on the initial length of the bonds (the C–B_C_ bond between carbon and boron atoms of the C–B_C_–C chain is the shortest one, see Fig. 1a, right panel; Fig. 1d, bottom panel; Fig. 1b, right panel). Points corresponding to inter-icosahedral bonds in α-B (green circles in Fig. 4) drop on the same line: initially shorter B_P_–B_P_ bonds contract slower than the initially longer B_E_–B_E_ contacts. All points corresponding to the intra-cluster B-B contacts, i.e. those involved in formation of the polycentral bonds of the B_12_ *closo*-cluster, for both B_13_C_2_ and α-B (orange squares and red circles, correspondingly, in Fig. 4) appear in a very compact area in Fig. 4, indicating similar rates of contraction and their similar lengths at ambient conditions. Why then the B_12_ icosahedra in B_13_C_2_ and α-B undergo such a dramatically different volume reduction (18.1 *vs* 14.5%) when their crystals are compressed to 60 GPa? Purely geometrical consideration is simply not appropriate. One must take into account types of bonding in each of the solids.

**Figure 5:**
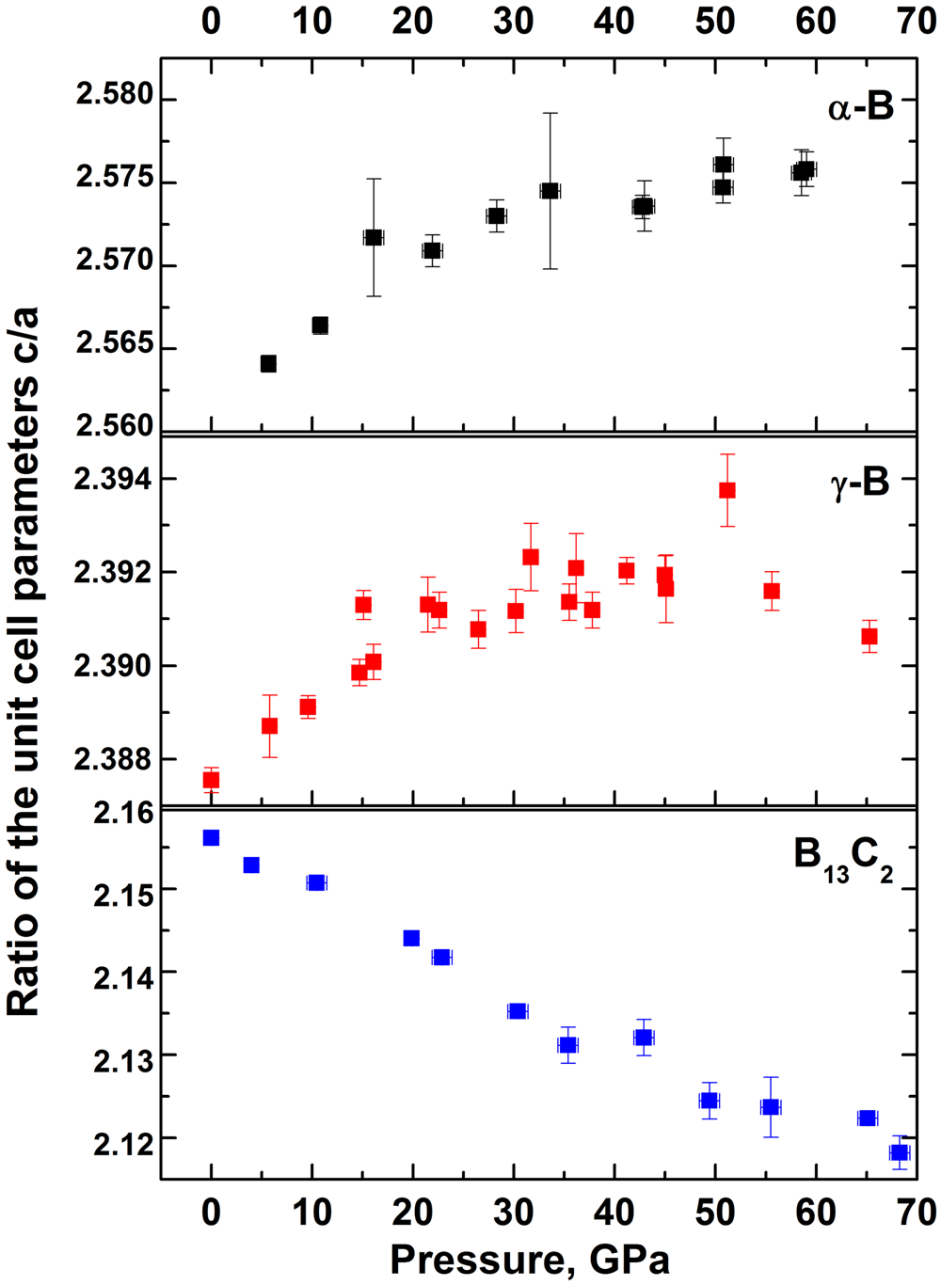
Evolution of the ratios of the unit cell parameters *c/a* of α-B and B_13_C_2_ compared to that of *c′/a′* of γ-B. For α-B the data are taken from Chuvashova *et al*.^25^ for γ-B from Zarechnaya *et al*.^9^.

As already mentioned, due to recent experimental electron-density studies of boron allotropes and boron carbide^13, 27, 28^, the validity of the Wade-Jemmis model (see introduction) was demonstrated for α–B^27^, γ–B^28^, and B_13_C_2_^13^. In all these solids, the molecular-orbital-type bonding on the icosahedral clusters leaves for *exo*-cluster bonding twelve *sp* hybrid orbitals perpendicular to the surface of the clusters. Thus, in terms of bonding, we can consider B_12_ *closo*-clusters to be similar in these three substances. Concerning the inter-cluster bonds, they were found to be very different in α–B^27^, γ–B^28^, and B_13_C_2_^13^. In α–B and B_13_C_2_ the B_P_–B_P_ bonds connecting polar B_P_ atoms (those between neighboring close-packed layers of B_12_ icosahedra) are strong covalent *2e2c* bonds (see Fig. 1a–c, left and right panels; Fig. 1d, top and bottom panels). The B_E_–B_E_ contacts in α–B^27^ are very flexible, because they are a part of relatively weak *2e3c* B_E_–B_E_–B_E_ bonds (shown by brown triangles in Fig. 1). Instead of these weak bonds, in the same positions (octahedral voids of the *ccp*) B_13_C_2_ possesses three strong *2e2c* C–B_E_ bonds, which are additionally strengthened by the *3e3c* bonds of the C–B_C_–C chains, which impose supplementary negative pressure upon the surroundings, as described in ref. 13. Now it is clear that the similar contraction of the crystals of α–B and B_13_C_2_ (ca. 18%) happens in B_13_C_2_ on the expense of the icosahedra, which are set into the very rigid surrounding of strong *2e2c* and even stronger *3e3c* bonds, but in α–B on the expense of weak *2e3c* bonds. This conclusion is justified by the behavior of the unit cell parameters ratio *c/a* in α–B and B_13_C_2_ upon compression: in α–B the *c/a* ratio increases with pressure, but it decreases in B_13_C_2_ (Fig. 5). In the both cases, above ca. 40 GPa a clear tendency to the leveling is observed that reflects the more homogeneous compression in the both directions at further pressure increase.

In the light of the consideration made for α–B and B_13_C_2_ above, it is not a surprise now that the volume reduction of B_12_ icosahedra in γ–B (16.9%) is intermediate between those in α–B and B_13_C_2_ in the same pressure range. As shown in ref. 28, in γ–B there is a broad diversity of inter-icosahedral bonds and those involving the boron dumbbell (Fig. 1a–d, middle panels): three different kinds of strong *2e2c* bonds (B3-B3, B2-B5, and B5-B5); for consistency, we follow here the atoms and bonds notations introduced for γ–B in refs 9 and 28. Although initially B5-B5 is slightly longer than B3-B3 and B2-B5, it contracts slightly slower than the latter two. The presence of two types (*2e3c* and *1e2c*) of polar-covalent bonds (*2e3c* B4-B4-B5, involving two boron atoms of one icosahedron and one atom of a dumbbell, and *1e2c* B1-B4, involving atoms of neighboring icosahedra at a distance slightly shorter than that between the B_E_ atoms in α–B) makes the rigidity of the inter-icosahedral space in γ–B to be intermediate between those in α–B and B_13_C_2_ (see Fig. 1). To compare the pressure evolution of the *c/a* ratio in α–B and B_13_C_2_ with the evolution of the corresponding *c′/a′* ratio in the structure of γ–B (*c′* is in the direction perpendicular to the close-packed layers and *a′* - within the layer), we expressed the *c′* and *a′* through the *a*, *b* and *c* parameters of the orthorhombic unit cell of γ–B and plotted the *c′/a′* ratio *vs* P in Fig. 5. As seen, the *c′/a′* ratio in γ–B, similarly to what is observed in α–B, first slightly increases, then saturates. Supplementary Figure S1 shows the pressure evolution of the normalized *c/a* ratios for all of these three solids: the *c′/a′*(P) curve of γ–B indeed takes an intermediate position between the *c/a*(P) curves of α–B and B_13_C_2_. Thus, the compressional behavior of γ–B is likely controlled by the balance between the contraction of icosahedra and the inter-icosahedral bonds. Figure 4 confirms this interpretation: the lengths of intra-icosahedral contacts in γ–B at ambient pressure are quite similar to those of α–B and B_13_C_2_ (see positions of purple triangles in Fig. 4), whereas rates of their contraction under pressure vary considerably (see their scatter within the black rectangular outlining the intra-icosahedral bonds in Fig. 4). Some of them are as compliant as inter-icosahedral contacts (B1-B4 and B5-B5), whereas others are almost as stiff as *2e2c* bonds inter-icosahedral and dumbbell bonds of γ–B itself and *2e2c* bonds of boron carbide.

Coming back to the difference in the compressibility of icosahedra in B_4_C^10^ and stoichiometric boron carbide B_13_C_2_, one should take into account different chemical composition, i.e. the boron to carbon ratio, of these two carbides. It is established that boron carbide exists as a single-phase material with a wide homogeneity range of carbon content, from ~7 at. % (B_14_C) to ~20 at. % (B_4.3_C), realized by the substitution of boron and carbon atoms for one another within both the icosahedra and intericosahedral chains^16^. Proposed stoichiometries are based on two extreme models, B_12_ (CBB) on the boron-rich and B_11_C(CCC) on carbon-rich ends. The location of C atoms in the crystal structure has been a long-standing debate^10, 14–24^, as it was difficult to clarify on the basis of powder diffraction data, or even single-crystal diffraction data of insufficient quality. Spectroscopic methods cannot directly give the localization of atoms, as the interpretations of spectra depend on models involved, the spectral range considered, and many other factors which introduce uncertainties.

For their “nearly stoichiometric boron carbide B_4_C” Dera *et al*.^10^ suggested the presence of 85 atomic % boron and 15 atomic % carbon in the B_P_ positions in B_11_C icosahedra. The presence of carbon likely has to lead to an increase of the rigidity of the icosahedron *closo*-cluster of B_4_C due to the higher electronegativity of carbon compared to boron. Indeed, the volume reduction of the B_11_C icosahedron (13%)^10^ appears to be smallest compared to B_12_ icosahedra in boron allotropes α–B (14.5%) and γ–B (16.9%) (see Table 2). The stoichiometric boron carbide B_13_C_2_ studied in the present work has been proven to contain carbon only in the C-B-C chains^13^. The refinement of the site occupancies for B_C_ and C atoms at all pressure points (except the point at 4 GPa, where R_int_ is relatively high and there is no meaning to refine additional structural parameters) gives the value of 1.000 (2). Thus, there is no any sign of vacancies in the C–B_C_–C triads. An attempt to substitute B_P_ and B_E_ atoms in icosahedra by carbon and refine the amount of C results in a full occupancy of the corresponding positions by boron atoms (within the uncertainty of 0.003). The B_12_ icosahedra in the studied boron carbide have a proven B_12_ composition (see also ref. 13 for extended discussion) and appear to be much more compliant than B_11_C icosahedra (Table 2); moreover, their compliance is similar to that of the whole structure. This observation gives additional evidence that the stoichiometric boron carbide B_13_C_2_ is a compound with true covalent bonding: B_12_ icosahedra do not play a role of “molecules”, their conventional separation is surely convenient for geometric presentation of the structure, but the compressional behavior of the stoichiometric boron carbide is governed by a complex interplay of both intra-cluster and inter-cluster bonds, as well as those involving C–B–C chains.

To conclude, in the present work we have studied the compressional behavior of the stoichiometric boron carbide B_13_C_2_ in the pressure interval up to 68 GPa. Our single-crystal synchrotron X-ray diffraction investigations revealed structural stability of the boron carbide in the studied pressure range. A comparison of the unit cell volume reduction with the reduction of the volume of the B_12_ icosahedron upon compression of B_13_C_2_ from ambient pressure to 60 GPa revealed their similarity. This confirms that the stoichiometric boron carbide B_13_C_2_ is a true covalent compound and does show neither ‘molecular-like’ nor ‘inversed molecular-like’ solid behavior upon compression, as previously disputed. Our analysis has shown that, in agreement with the modern understanding of bonding in α–B, γ–B, and B_13_C_2_ based on the experimental electron-density studies^13, 27, 28^, the compressional behavior of these boron allotropes and boron carbide depends on the types of bonding involved in the course of compression, so that the ‘effective compressibility’ of B_12_ icosahedra may vary in a broad range, from ca. 14% in α–B to ca. 18% in B_13_C_2_, as compared to ca. 18% of compression of the corresponding crystals.

## Methods summary

### Synthesis of crystals

Single crystals of B_13_С_2_ were synthesized at high pressures (8.5–9 GPa) and high temperatures (1873–2073 K) using the large-volume-press technique. The stoichiometric composition B_13_C_2_ was determined by single-crystal X-ray diffraction in agreement with energy-dispersive X-ray (EDX) analysis (B_6.51 (12)_C)^13^ and results of Laser Ablation ICP-MS (B_6.5(2)_C). By LA-ICP-MS boron, carbon, and trace element concentrations in the material were measured using a 193 nm ArF Excimer laser (GeolasPro, Coherent, USA) attached to an Elan DRC-e (Perkin Elmer, Canada) quadrupole mass spectrometer. The presence of impurities exceeding ppm level could be excluded. At ambient conditions the crystals of the stoichiometric boron carbide B_13_C_2_ are semitransparent and have a characteristic dark red or maroon color^13^. This optical property distinguishes them from crystals of non-stoichiometric or disordered boron carbide, which is described as black material.

### Diamond-anvil cell experiments

The BX90-type diamond anvil cells (DAC)^29^ made at Bayerisches Geoinstitut (Bayreuth, Germany) and diamonds with the culet diameters of 250 µm were used in high pressure experiments. Rhenium gaskets were squeezed between the diamonds to make an indentation with the thickness of 30 µm. Then in the center of indentations, round holes of 120 µm in diameter were drilled. The B_13_С_2_ crystals were placed into these chambers. Sizes of the crystals were about 10 × 10 × 15 µm^3^ and 20 × 15 × 10 µm^3^. Neon was used as a pressure transmitting medium. Ruby balls used for pressure calibration were placed into the pressure chamber.

### Single-crystal X-ray diffraction

Crystals with size of about 10 × 10 × 15 µm^3^ were selected for measurements in a DAC at ID27 at the European Synchrotron Radiation Facility (ESRF). Diffraction data were collected at 293 K using the Perkin Elmer XRD1621 flat panel detector. The monochromatic radiation had the wavelength of 0.37380 Å and the crystal-to-detector distance was 383 mm. Pressure in the cell was increased up to 68 GPa with a step of about 6 GPa. 160 frames in the omega scanning range of −40° to +40° were collected (0.5° scanning step size) with an exposure time of 1 s. Integration of the reflection intensities and absorption corrections were performed using CrysAlisPro software^30, 31^. The structure was refined in the anisotropic approximation for all atoms by full matrix least-squares using Jana2006 software^32^.

## Electronic supplementary material

Supplementary Information
