# Supplementary material for: Structural stability and mechanism of compression of stoichiometric B13C2 up to 68GPa
Source: Sci Rep. 2017 Aug 21;7:8969. doi: 10.1038/s41598-017-09012-8 (PMC5567096; doi:10.1038/s41598-017-09012-8)
Supplement: Supplementary file 1 — Supplementary Information [file 41598_2017_9012_MOESM1_ESM.pdf]

Supplementary Information for

Structural stability and mechanism of compression of stoichiometric $B_{13}C_2$ up to 68 GPa

Irina Chuvashova*, Elena Bykova, Maxim Bykov, Volodymyr Svitlyk, Leonid Dubrovinsky, Natalia Dubrovinskaia*

**Correspondence to:* *irina.chuvashova@gmail.com*, natalia.dubrovinskaia@uni-bayreuth.de

This PDF file includes
Supplementary Table S1
Supplementary Figure S1

Supplementary Table S1. Fractional atomic coordinates and equivalent isotropic displacement parameters for $B_{13}C_2$ at variable pressure. (Crystal 2 is designated as “cr2”). *Wyckoff positions are *18h* (x, $\bar{x}$, z) for both B_P and B_E atoms, *3a* (0,0, 0) for B_C atom and *6c* (0, 0, z) for C atom.

P, GPa	Atom*	x	z	U_{eq}, Å^2
4.0(5)	B_P	0.4401(3)	0.0527(2)	0.0028(10)
	B_E	0.5032(3)	0.1922(2)	0.0033(10)
	B_C	0	0	0.0091(19)
	C	0	0.1189(4)	0.0036(11)
10.0(5)	B_P	0.4404(3)	0.0522(2)	0.0101(10)
	B_E	0.5038(3)	0.1918(2)	0.0102(10)
	B_C	0	0	0.0123(17)
	C	0	0.1193(3)	0.0097(11)
20(1)	B_P	0.4401(3)	0.05192(19)	0.0070(7)
	B_E	0.5036(3)	0.19179(18)	0.0075(8)
	B_C	0	0	0.0120(15)
	C	0	0.1202(3)	0.0074(9)
23(1)	B_P	0.4396(6)	0.0519(4)	0.0156(15)
	B_E	0.5034(6)	0.1921(3)	0.0156(15)
	B_C	0	0	0.020(3)
	C	0	0.1206(6)	0.0162(18)
30(1) cr2	B_P	0.4406(2)	0.05175(19)	0.0029(9)
	B_E	0.5036(2)	0.1918(2)	0.0032(9)
	B_C	0	0	0.0071(15)
	C	0	0.1208(3)	0.0036(9)
35(1) cr2	B_P	0.4403(2)	0.05150(19)	0.0076(8)
	B_E	0.5032(2)	0.19180(17)	0.0075(8)
	B_C	0	0	0.0144(15)
	C	0	0.1212(3)	0.0088(9)
43(1) cr2	B_P	0.4401(2)	0.05114(19)	0.0074(8)
	B_E	0.5032(2)	0.19201(17)	0.0072(8)
	B_C	0	0	0.0147(15)
	C	0	0.1217(3)	0.0079(9)
49(1) cr2	B_P	0.4401(2)	0.05062(18)	0.0055(8)
	B_E	0.5033(2)	0.19167(18)	0.0061(8)
	B_C	0	0	0.0153(15)
	C	0	0.1226(3)	0.0075(9)
56(1) cr2	B_P	0.4402(2)	0.05059(18)	0.0041(8)
	B_E	0.5032(2)	0.19185(19)	0.0050(8)
	B_C	0	0	0.0151(16)
	C	0	0.1225(3)	0.0060(9)
65(1)	B_P	0.4402(4)	0.0504(2)	0.0044(11)
	B_E	0.5036(4)	0.1921(3)	0.0057(12)
	B_C	0	0	0.016(2)
	C	0	0.1232(4)	0.0069(12)
68(1) cr2	B_P	0.4398(3)	0.0509(2)	0.0026(9)
	B_E	0.5035(3)	0.1920(2)	0.0024(9)
	B_C	0	0	0.0153(18)
	C	0	0.1233(3)	0.0036(10)

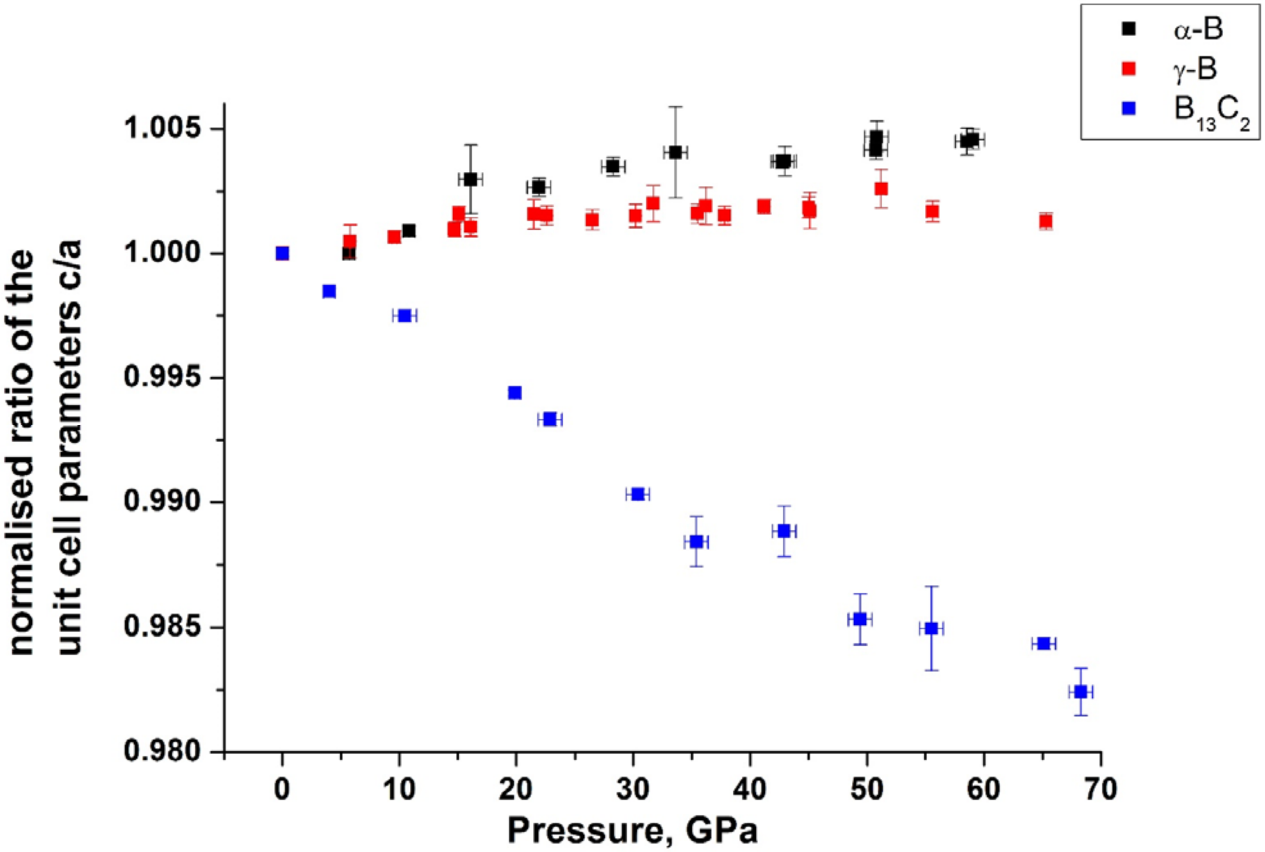

Supplementary Figure S1. Evolution of the normalized ratios of the unit cell parameters *c/a* of α-B and $B_{13}C_2$ compared to that of *c´/a´* of γ-B. For α-B the data are taken from Chuvashova et al.[25]; for γ-B from Zarechnaya et al.[9]
